# Synthesis, Characterization, and Osteoblastic Cell Culture of Poly(L-co-D,L-lactide-co-trimethylene carbonate) Scaffolds

**DOI:** 10.1155/2014/501789

**Published:** 2014-06-25

**Authors:** André Dutra Messias, Kelly Fernanda Martins, Adriana Cristina Motta, Eliana Aparecida de Rezende Duek

**Affiliations:** ^1^Department of Materials Engineering, Faculty of Mechanical Engineering, State University of Campinas, Rua Mendeleyev 200, 13083-860 Campinas, SP, Brazil; ^2^Laboratory of Biomaterials, Faculty of Medical Sciences and Health, Pontifical Catholic University of São Paulo, Rua Joubert Wey 290, 18030-070 Sorocaba, SP, Brazil; ^3^Federal University of Sao Carlos, Rodovia João Leme dos Santos, Km 110, SP-264, Itinga, 18052-780 Sorocaba, SP, Brazil

## Abstract

Lactide-based polymers have been widely investigated as materials for tissue engineering. However, characteristics such as low flexibility and elongation tend to limit particular applications, although these can be enhanced by adding plasticizers such as trimethylene carbonate (TMC) to the polymer chain of the copolymer poly(L-lactide-co-D,L-lactide) (PLDLA). The aim of this work was to synthesize and characterize a terpolymer of L-lactide, D,L-lactide, and TMC. The polymers were synthesized from 30% TMC by bulk polymerization and resulted in an average molar mass >10^5^ g/mol. Thermal investigation of PLDLA-TMC showed a decrease in the glass transition and onset temperatures compared to PLDLA. PLDLA-TMC scaffolds stimulated the proliferation and normal phenotypic manifestations of cultured osteoblasts. These results show that it was possible to produce a terpolymer from L-lactide, D,L-lactide, and TMC. Scaffolds of this terpolymer had important characteristics that could be useful for applications in bone tissue engineering.

## 1. Introduction

Bone has a limited capacity for regeneration, especially in cases involving large losses as a result of complicated fractures caused by trauma, disease, birth defects, bone tumor removal, joint replacement, spinal fusion, or periodontal diseases. Bone loss is a major challenge for modern medicine, particularly since current techniques, such as metallic implants and autologous bone grafts, have significant drawbacks. There is therefore a need to develop novel therapeutic approaches such as bone tissue engineering [1, 2].

Tissue engineering seeks to create an alternative means of repairing bone injuries by combining a scaffold, cells, and growth factors to produce tissue substitutes capable of restoring or replacing lost tissues and organs. The approaches used include cell therapies, induction of tissue/organ regeneration by biologically active molecules, and the transplantation of tissues grown* in vitro* [2, 3]. An underlying principle in this approach is that biomaterial or cell-biomaterial constructs degrade concomitantly with tissue regeneration.

Poly(*α*-hydroxyacids) are biodegradable, resorbable materials with good biocompatibility and these features make them preferred candidates for tissue engineering. The lactide monomers have different chiral molecules. Among lactide-derived polymers, only poly(L-lactide) and poly(D,L-lactide) have been extensively investigated as biomaterials. The biological properties of the copolymer poly(L-co-D,L lactide) (PLDLA) remain poorly known whereas poly(L-lactide) (PLLA) is a semicrystalline polymer with a high modulus and strength. PLLA is known for its increasing crystallinity during degradation which can generate an intense inflammatory response [4]. In contrast, poly(D,L-lactide) (PDLLA) is an amorphous polymer characterized by the random distribution of the two isomeric forms along the polymer chain [5]. PLDLA is also amorphous and has mechanical features similar to those of PLLA without the inconvenience of long degradation times and high crystallinity.

The brittle nature of poly(lactides) requires modification in order to be useful as biomedical materials [6]. The need for biodegradable elastomeric polymers for medical implants and porous scaffolds in tissue engineering has been documented in recent years [7–9]. The suitability of poly trimethylene carbonate (TMC) for the preparation of biomedical implants has also been evaluated [10]. Poly(TMC) has a very low modulus and tensile strength and its poor mechanical properties have discouraged any practical application other than as a component of copolymers and blends. Poly(TMC) has been used as a softening unit in stiff and brittle polymers such as lactide [7, 11, 12] and glycolide [13].

Scaffolds are implants or injections that are used to deliver cells, drugs, and genes into the body. Different forms of polymeric scaffolds for cell/drug delivery are available, for example, typical three-dimensional porous matrices, nanofiber matrices, thermosensitive sol-gel transition hydrogels, and a porous microsphere. A scaffold provides a suitable substrate for cell attachment, cell proliferation, differentiated function, and cell migration [14].

Porous bioresorbable polymers have been widely used as scaffolds in tissue engineering. Macroporous structures are desirable in many cases to facilitate cell seeding, infiltration of fluids, vascularization, and tissue ingrowth. In this context, several techniques have been developed to create porous scaffolds, including solvent casting/particulate leaching, fiber bonding, gas foaming, phase inversion, and solvent removal by freeze-drying. The morphology and properties of the resulting scaffolds depend largely on the fabrication process used [15].

Polymeric scaffolds should be designed to facilitate cell distribution and guide tissue regeneration in three dimensions [16]. Among the different methods for producing scaffolds, solvent casting followed by precipitation or particulate leaching is one of the most straightforward methods [17, 18]. Particulate leaching is a general procedure that is used to obtain porous scaffolds in solvent casting methods [19, 20].

In this study, scaffolds of PLDLA and PLDLA-TMC were prepared by solvent casting/particulate leaching using saccharose crystals as the porogen. The copolymers synthesized were characterized by physical and chemical methods and the thermal properties of the scaffolds were investigated. The usefulness of the scaffolds for stimulating osteoblast adhesion and proliferation* in vitro* was also assessed.

## 2. Materials and Method

### 2.1. Poly(L-co-D,L-lactide-co-trimethylene carbonate) Synthesis

Polymerization was carried out using L-lactide (Purasorb L, Purac, Netherlands), D,L-lactide (Purasorb DL, Purac), and trimethylene carbonate (TMC, 1,3-dioxane-2-one; Boehringer Ingelheim Pharma, Germany) in 70 : 30 : 0 and 50 : 20 : 30 monomer ratios catalyzed by Sn(Oct)_2_ at 130°C for 48 h in a vacuum ampoule (INPI patent requisition, protocol number 020110049277). The polymers were subsequently purified by dissolution in chloroform and precipitation in methanol.

### 2.2. Physicochemical Characterization of the Synthesized Polymer

#### 2.2.1. Nuclear Magnetic Resonance (NMR)

Nuclear magnetic resonance (NMR) spectra were obtained with a Bruker 250 spectrometer operated at 300 MHz and 75 MHz for ^1^H and ^13^C, respectively, at room temperature. The polymer samples (10% w/v) were dissolved in deuterated chloroform (CDCl_3_) and tetramethylsilane was used as the reference standard.

#### 2.2.2. Fourier Transform Infrared (FTIR) Spectrometry

Fourier transform infrared (FTIR) spectrometry was carried out using a Perkin Elmer FT-IR Spectrum One spectrometer in the range of 4000–650 cm^−1^ after casting the polymer from chloroform solution onto sodium chlorite pellets.

#### 2.2.3. Gel Permeation Chromatography (GPC)

Gel permeation chromatography (GPC) analyses were carried out using a Waters instrument (1525 binary HPLC pump and 2414 refractive index detector, Waters Corporation, USA) equipped with Waters Styragel columns (HR 4E and HR 5E). Tetrahydrofuran was used as the mobile phase at a flow rate of 1 mL/min. The columns and detector were maintained at 30°C and 40°C, respectively.

### 2.3. Scaffold Fabrication, Morphology, and Thermal Properties

#### 2.3.1. Scaffold Fabrication

The polymer scaffolds were prepared by the solvent casting/particulate leaching method. Initially, the polymer was dissolved in chloroform (10% w/v) for 4 h. After complete dissolution, a 70% (w/v) solution of ground, sieved sucrose crystals (>250 *μ*m) was added. The mixture was then cast in silicone molds with cylindrical cavities (7 mm in diameter and 10 mm high) and the solvent was allowed to evaporate overnight at room temperature. Sucrose particles were removed by immersion in a 1% (w/v) aqueous solution of polyvinyl alcohol in order to reduce the surface tension between the polymer and water. Finally, the scaffolds were dried in a vacuum and sliced to form disks ~4 mm high.

#### 2.3.2. Scanning Electron Microscopy (SEM)

Morphological analysis of the scaffolds was carried out by scanning electron microscopy (SEM) using a JEOL JXA-840A scanning electron microscope after gold coating (Balzer SCD 050) of the samples. Some samples were immersed in liquid nitrogen and fractured in order to examine the internal structure of the scaffold.

#### 2.3.3. Scanning Differential Calorimetry (DSC)

Scanning differential calorimetry (DSC) measurements were carried out on a DSC 2920 thermal analyzer (TA Instruments, USA). Polymeric samples were hermetically sealed in aluminum pans and heated at 200°C to destroy the thermal history of the polymers. Subsequently, the samples were exposed to temperatures ranging from −50°C to 200°C at increments of 10°C min^−1^, in an inert nitrogen atmosphere flushed at 5 mL min^−1^.

#### 2.3.4. Thermogravimetric Analysis (TGA)

The thermal stability of the scaffolds was tested in an STA 409C thermogravimetric analyzer (Netzsch) in which the samples were heated at 10°C min^−1^ up to 500°C, in an inert argon atmosphere.

### 2.4. Osteoblast Cell Culture on Scaffolds

#### 2.4.1. Cell Culture

Human osteoblast-like SaOS-2 cells were purchased from the Rio de Janeiro Cell Bank (Rio de Janeiro, RJ, Brazil) and cultured in McCoy's 5A modified medium (CULTILAB, Brazil) containing L-glutamine, HEPES, 15% fetal bovine serum (FBS—CULTILAB, Brazil), 50 mg of getamicin/L (Invitrogen), and 2.5 mg of amphotericin B/L (Invitrogen) in a humidified 95% air/5% CO_2_ incubator, at 37°C. The medium was changed every 3-4 days. Cells were detached by treatment with a trypsin/EDTA solution (CULTILAB, Brazil).

The scaffolds were sterilized by immersion in 70% ethanol for 1 h and repeatedly washed with deionized water. Before cell culture, sterilized scaffolds were maintained in McCoy's 5A medium for ~24 h.

A cell suspension (1 × 10^4^ cells/well in 200 *μ*L of growth medium) was seeded onto scaffolds of PLDLA-TMC and PLDLA in 96-well plates (TPP, Switzerland) and cultured for 1, 3, 7, and 14 days. Control cells were cultured in wells without scaffolds. The long-term culture clarifies how the scaffolds can sustain the cell proliferation and also allows the observation of the cell differentiation.

#### 2.4.2. MTT Assay

To evaluate the mitochondrial activity of the seeded cells, that is, cell viability on the scaffolds during culture, the samples and controls were washed 2-3 times with McCoy' 5A medium without serum and then incubated with 200 *μ*L of 3-(4,5-dimethyl-2-thiazolyl)-2,5-diphenyl-2H-tetrazolium bromide (MTT—Sigma-Aldrich) in McCoy's medium (0.5 mg mL^−1^). The plates were incubated for 4 h in a cell incubator at 37°C, after which the MTT solution was replaced with 200 *μ*L of dimethylsulphoxide (DMSO—Sigma-Aldrich). After 10 min, 100 *μ*L aliquots were transferred to empty wells and the absorbances were measured in a microplate reader (Elx-800-UV, Bio-Tek Instruments) at 570 nm and 650 nm.

#### 2.4.3. ALP Assay

Alkaline phosphatase (ALP) produced by SaOS-2 was quantified as an osteoblast marker using an ALP kit (Labtest, Brazil). Briefly, 10 *μ*L aliquots of culture supernatant were collected and added to 100 *μ*L of* p*-nitrophenol phosphate solution (12 mM* p*-nitrophenol phosphate, 10 mM phenol, and 1.6 mM EDTA buffered to pH 10.4, 0.96 mM zinc sulfate, 2 mM magnesium acetate, and 6.4 mM sodium azide). The reagent was run for 2 min at 37°C and the changes in absorbance were measured in a microplate reader at 415 nm and 650 nm.

## 3. Results and Discussion

### 3.1. Physicochemical Characterization of the Synthesized Polymer

Poly(L-co-D,L-lactide-co-trimethylene carbonate) was synthesized by bulk polymerization via ring-opening of cyclic dimer monomers at 130°C for 48 h in a vacuum atmosphere.  Table 1 shows molar mass of terpolymer measured by GPC. The catalyst used was stannous octoate (Sn(Oct)_2_) in a monomer/catalyst ratio of 5,000 (w/w). Previous studies demonstrated that this monomer/catalyst ratio produced higher yields and greater molar mass values [21].

**Figure 1:**
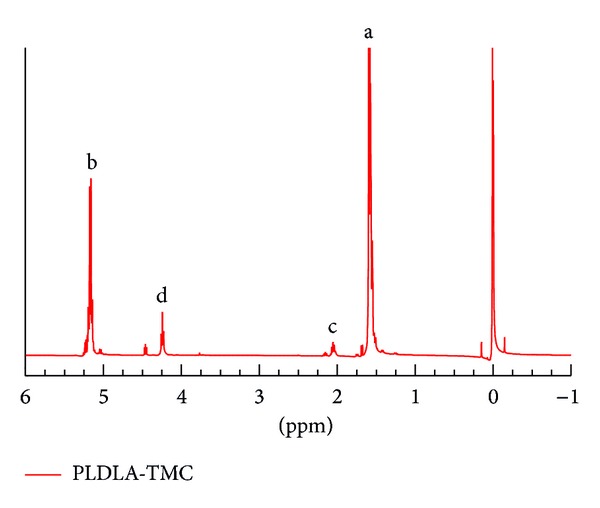
^1^H NMR spectra of PLDLA-TMC.

Figure 1 shows the ^1^H-NMR spectra of PLDLA-TMC. The signals found in the terpolymer ^1^H-NRM were practically the same as those for the poly(L-lactide-co-D,L lactide) copolymer and differed at only two points that are characteristic of TMC, that is, *δ* 2.05 ppm (CH_2_-TMC) and *δ* 4.24 ppm (OCH_2_-TMC) [22]. In the copolymer spectrum (data not shown), a multiplet at 5.12–5.24 ppm was assigned to the CH (b) proton, while the quartet at 1.55–1.59 ppm was assigned to the protons CH_3_ (a) [23]. In the case of PLDLA-TMC, the triplet at 2.05 ppm was assigned to the protons CH_2_ (c), while the triplet at 4.24 was assigned to OCH_2_ (d).

**Figure 2:**
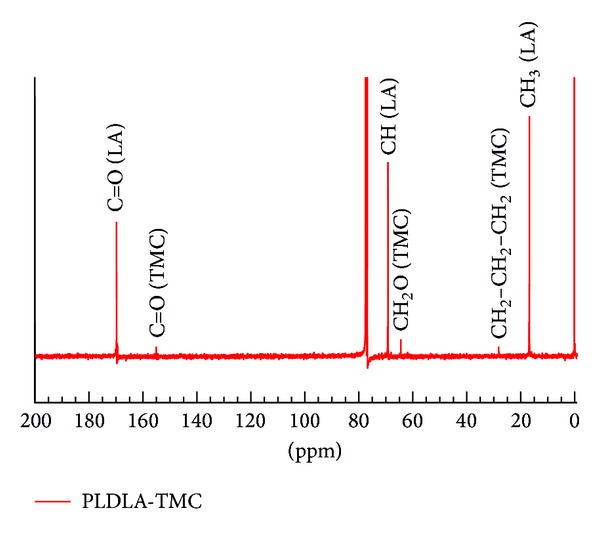
^13^C NMR spectrum of PLDLA-TMC.

The ^13^C-NMR spectrum (Figure 2) showed lactide units at 16.6 (CH_3_), 69.0 (CH), and 169.6 (C=O) ppm and TMC units at 27.8 (CH_2_–CH_2_–CH), 61.8–64.7 (CH_2_O), and 154.8 (C=O) ppm. These chemical shifts agreed with the findings of Matsumura et al. [24] who synthesized low molar mass (21,000 g mol^−1^) lactide/TMC copolymers using a lipase-catalyzed reaction.

**Figure 3:**
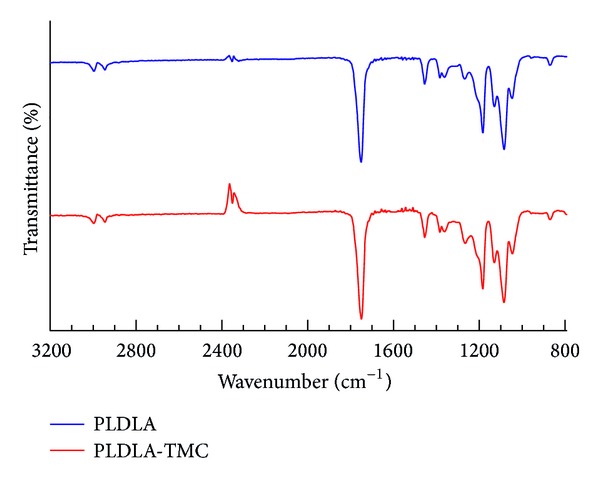
FT-IR spectra of PLDLA and PLDLA-TMC.

Figure 3 represents PLDLA and PLDLA-TMC infrared spectra and shows no difference when TMC is present. The strongest peaks are found at 1181 and 1746 cm^−1^ corresponding to C–C bonds and carbonyl axial deformation, respectively.

### 3.2. Scaffold Construction, Morphology, and Thermal Properties

**Figure 4:**
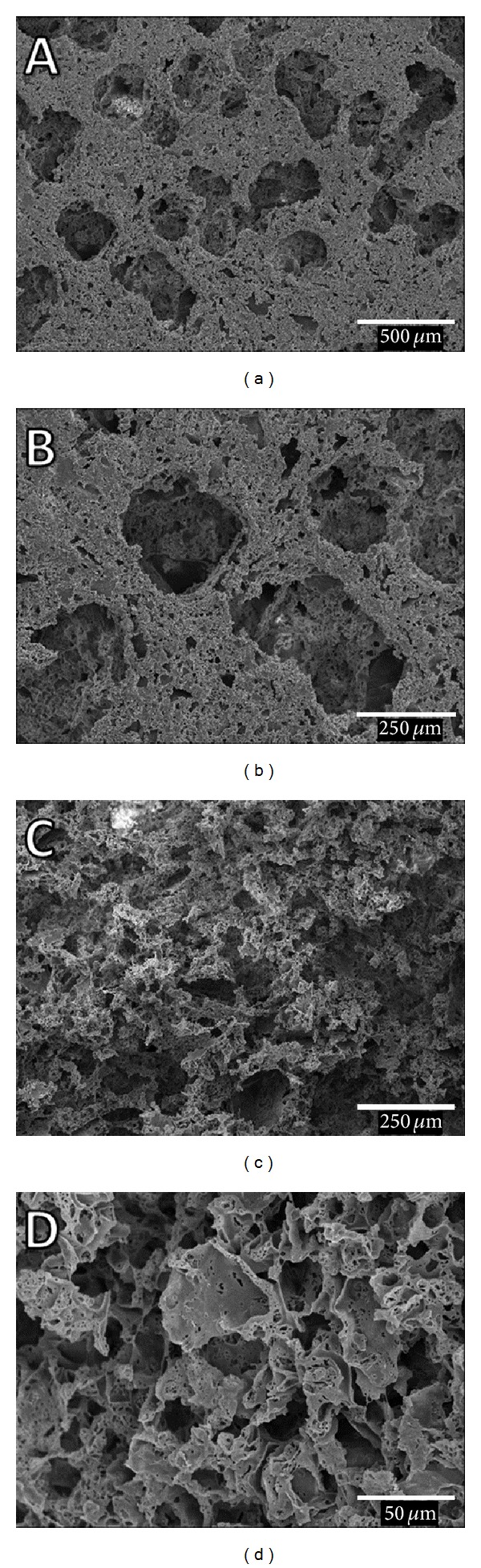
SEM of PLDLA-TMC scaffolds showing the external (a, b) and inner (c, d) surfaces. Inner views of the scaffolds were obtained after cryofracture.

SEM revealed the porous morphology of the scaffold surface (Figures 4(a) and 4(b)) and interior (Figures 4(c) and 4(d)). The pores were irregular in shape and consisted of a mixture of large pores (~250–500 *μ*m in diameter) and small pores (<100 *μ*m). Inner side of the scaffold is highly porous compared to the scaffold surface (Figures 4(c) and 4(d)). Scaffold porosity and pore size are important features when evaluating biomaterial properties for tissue engineering [25]. An adequate pore size allows cell invasion and tissue ingrowth to surround the local implant while maintaining adequate nutrient transfer; however, there is some controversy regarding the best pore that favors new bone formation and growth [26–28]. The porosity and pore structure can be controlled by salt weight, particle size, and polymer fraction [25].

**Figure 5:**
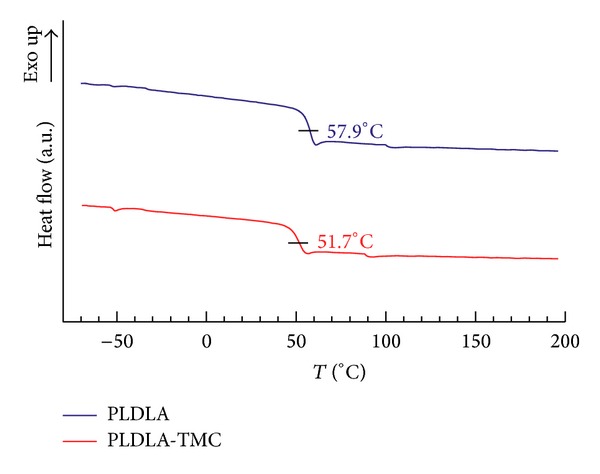
DSC thermograms showing the glass transition temperature of PLDLA and PLDLA-TMC scaffolds.

The results for the thermal characterization of the scaffolds are shown in Figures 5 (DSC) and 6 (TGA). None of the polymeric scaffolds showed evidence of crystallinity. PLDLA-TMC was amorphous and DSC thermograms showed a decrease in the glass transition temperature (*T*_g_) of PLDLA-TMC (51.7°C) compared to PLDLA (57.9°C) (Figure 5). Similar tendencies were observed for the *T*_g_ values for poly(L-lactide-co-TMC) and poly(D,L lactide-co-TMC) [24]. Davachi et al. [29] showed that an increase in the TMC content of poly(lactide-TMC-glycolide) resulted in a decrease in the glass transition temperature. Since the crystalline phase is poorly accessible to water and other permeants, the presence of crystallinity reduces polymer permeability and the rate of biodegradation by chain scission because of a decrease in accessible hydrolysable bonds. With regard to the use of these polymers for biomedical applications, it is worth remembering that crystalline debris formed during degradation may cause an undesired late inflammatory response, thereby negatively influencing tissue growth and normal function [30]. Hence, little or no crystallinity is desirable [31].

**Figure 6:**
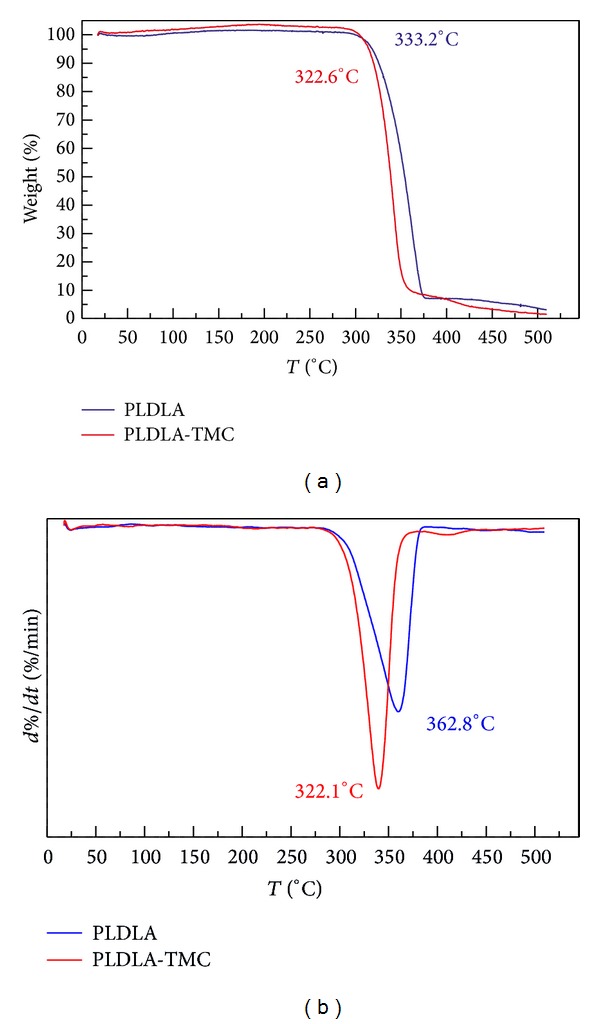
TGA thermograms of PLDLA and PLDLA-TMC scaffolds.

The thermal stability of PLDLA-TMC was measured with a thermogravimetric analyzer (TGA).  Figure 6 shows typical TGA traces of weight loss as a function of temperature.

This analysis showed that both polymers decomposed in a single-stage degradation and that the addition of TMC reduced the polymer thermal stability; that is, the addition of TMC to L/D,L-lactide resulted in greater degradation of the polymer. The onset temperature of PLDLA decreased from 333.2°C to 322.6°C when TMC was present. The maximum decomposition temperature of PLDLA was 362.8°C, whereas that of PLDLA-TMC was 342.1°C.

### 3.3. Osteoblast Cell Culture on Scaffolds

**Figure 7:**
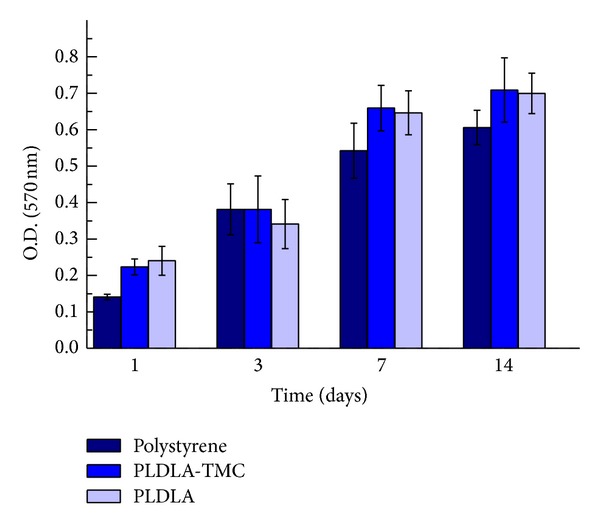
Viability of SaOS-2 osteoblasts grown on scaffolds. The values are the mean ± SD (*n* = 6).

The MTT assay showed that the viability of SaOS-2 osteoblasts increased with time (Figure 7), an indication of cell proliferation on the porous scaffolds. The presence of TMC in PLDLA did not affect cell viability compared to scaffolds containing only PLDLA. After 1 and 7 days, cells grown on PLDLA and PLDLA-TMC scaffolds showed greater viability than those grown in control wells (*P* < 0.05). After 14 days, only cells grown on PLDLA-TMC scaffolds had greater viability than control cells (*P* < 0.05). SaOS-2 proliferation ceased at 7 days since the viability was similar at 7 and 14 days.

**Figure 8:**
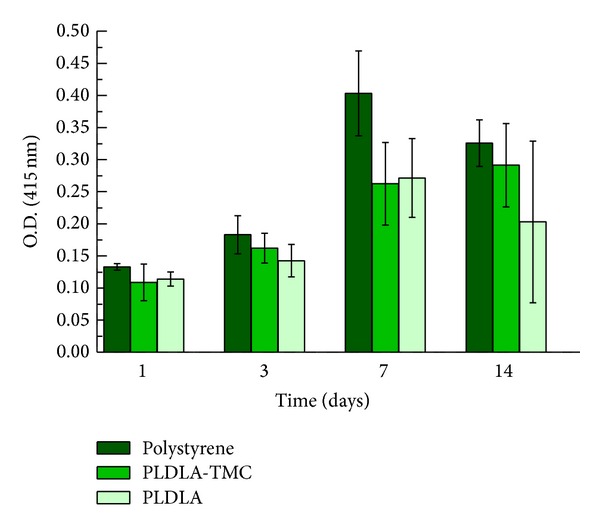
Alkaline phosphatase activity of SaOS-2 on scaffolds. Values are expressed as means ± SD, *n* = 6.

Early osteoblastic differentiation was demonstrated by the ALP activity of SaOS-2 cells (Figure 8). There was no significant difference in the ALP activities of PLDLA and PLDLA-TMC scaffolds. The ALP activity increased from days 1 and 3 to days 7 and 14 (*P* < 0.05).

Stein and Lian [32] identified two stages of osteoblast differentiation, namely, proliferation during the first 7–14 days and the secretion of extracellular matrix proteins and production of early differentiation markers, such as ALP, from day 7 onwards. ALP belongs to a group of membrane-bound glycoproteins involved in the deposition of minerals on extracellular matrix molecules [33].

## 4. Conclusion

Poly(L-co-D,L-lactide-co-trimethylene carbonate) (PLDLA-TMC) was synthetized by ring-opening bulk polymerization of the monomers, using stannous octanoate as catalyst. Thermal analysis showed that the presence of TMC reduced the glass transition temperature and decreased the polymer thermal stability. The scaffolds had a porous morphology that was conducive to cell growth, as shown in the viability and proliferation of SaOS-2 osteoblasts. The detection of alkaline phosphatase activity demonstrated that the cells retained their osteoblast phenotype. Thus, PLDLA-based devices are potentially useful for bone tissue engineering.

## Figures and Tables

**Table 1:** Molar mass of terpolymer.

% TMC	Mw (g/mol)	Mn (g/mol)	PDI
20% TMC	127.630	69.694	1,7
30% TMC	126.577	60.451	2,0

Mw = weight average molecular weight; Mn = number average molecular weight; PDI = polydispersity index.
